# Interleukin-1β induced Stress Granules Sequester COX-2 mRNA and Regulates its Stability and Translation in Human OA Chondrocytes

**DOI:** 10.1038/srep27611

**Published:** 2016-06-08

**Authors:** Mohammad Y. Ansari, Tariq M. Haqqi

**Affiliations:** 1 Department of Anatomy and Neurobiology, North East Ohio Medical University, Rootstown, Ohio, USA

## Abstract

Enhanced and immediate expression of cyclooxygenase-2 (COX-2) mRNA is observed in IL-1β-stimulated OA chondrocytes but the synthesis of protein found significantly delayed. Here we investigated the role of stress granules (SGs), ribonucleoprotein complexes that regulate mRNA translation, in the delayed translation of COX-2 mRNAs in IL-1β-stimulated OA chondrocytes. Stimulation of human chondrocytes with IL-1β activated the stress response genes and the phosphorylation of eIF2α that triggered the assembly of SGs. Using combined immunofluorescence staining of SGs markers and COX-2 protein, RNA fluorescence *in situ* hybridization and RNA immunoprecipitation, the COX-2 mRNAs were found sequestered in SGs in IL-1β-stimulated OA chondrocytes. No increase in COX-2 protein expression was observed during the persistence of SGs but enhanced expression of COX-2 protein was noted upon clearance of the SGs. Inhibition of SGs clearance blocked COX-2 mRNA translation whereas blocking the assembly of SGs by TIA-1 depletion resulted in rapid and increased production of COX-2 and PGE2. Our findings show for the first time assembly of SGs and sequestration of COX-2 mRNAs in human OA chondrocytes under pathological conditions. Post-transcriptional regulation of COX-2 mRNAs translation by SGs indicates a role in IL-1β-mediated catabolic response that could be therapeutically targeted in OA.

Osteoarthritis (OA) is a chronic degenerative joint disease in which the articular cartilage is progressively degraded leading to the loss of joint function. Non-classical inflammation is an integral component of the disease pathology and high levels of inflammatory cytokines and other mediators of inflammation have been detected in synovial fluid from OA patients^1^,^2^. Interleukin-1β (IL-1β), a pro-inflammatory cytokine, is released by activated synoviocytes, chondrocytes and invading macrophages in inflamed joints and plays a pivotal role in the pathogenesis of OA^3^,^4^. Increased IL-1β levels in synovial fluid of OA patients have been detected and the cytokine is known to activate signaling pathways in chondrocytes leading to the production of catabolic mediators such as prostaglandin E2 (PGE_2_) that play a significant role in cartilage matrix degradation and joint dysfunction^4^. Cyclooxygenase-2 (COX-2) is an immediate-early response gene, is not constitutively expressed in most cell types and is a rate-limiting enzyme in the production of PGE_2_ and other prostanoids. At very low levels, PGE_2_ may have anabolic functions in cartilage, but the nano- to micromolar concentrations produced by OA tissues are predominantly catabolic, which can lead to increased MMP-13 production and inhibition of proteoglycan synthesis^5^,^6^. COX-2 expression is tightly regulated through both transcriptional and post-transcriptional mechanisms, and this allows for rapid and increased prostaglandin synthesis when required^7^. In published studies^8^ and in our unpublished studies, we observed that although COX-2 mRNA was rapidly induced in human OA chondrocytes stimulated with IL-1β, COX-2 protein was detected several hours later. However, the reason for this delay in COX-2 mRNA translation is unclear.

Stress granules (SGs) are ribonucleoprotein complexes that allow the cell to adapt to various situations and to respond adequately to stress^9^. SGs contain translationally stalled mRNAs, associated pre-initiation factors and specific RNA binding proteins (RBPs)^10^. In addition to translation initiation factors, SGs contain many other proteins that are involved in RNA metabolism, including RBPs HuR, TIA-1, TTP, TIAR and PABP1, helicases and deacetylases^11^,^12^,^13^. TIA-1, HuR and TTP bind selectively to mRNAs with high ARE content and recruit them to SGs. Macrophages lacking TIA-1 or TTP secrete more tumor necrosis factor alpha (TNFα) protein than controls without affecting the mRNA expression level^14^. However, TIA-1 deletion has no effect on the expression of TNFα in lymphocytes^15^. Interestingly, TIA-1 functions as translational silencer for TNFα while HuR enhances TNFα mRNA half-life and its translation^16^. But how the two proteins function in coordination with each other in the cell and what is the common factor important for their coordinated function is not known. The role of TIA-1 or HuR proteins in the posttranscriptional gene regulation during the development and progression of OA has not been fully explored. In addition the assembly and role of SGs in gene regulation in chondrocytes under pathological conditions has also not been studied.

In this study, we demonstrate that IL-1β stimulated OA chondrocytes exhibited high levels of GRP78, a marker for ER stress, coupled with the expression and phosphorylation of eIF2α that is known to trigger the assembly of SGs. Assembly of SGs in IL-1β- stimulated OA chondrocytes served to capture and stalling of COX-2 mRNAs translation and also extended the half-life of COX-2 mRNAs. Our results also show that translocation and localization of COX-2 mRNAs to SGs was principally mediated by the RNA binding protein HuR. We also demonstrate that the appearance of COX-2 protein correlated with the disassembly of SGs in cytokine-stimulated OA chondrocytes and that blocking the clearance of SGs further delayed the expression of COX-2 protein and abolishing the assembly of SGs by TIA-1 depletion resulted in early and increased expression of COX-2 protein as well as PGE_2_ production. Although sequestered COX-2 mRNAs were principally associated with HuR in OA chondrocytes, knockdown of TIA-1 abolished SGs assembly and prevented the localization of COX-2 mRNAs to SGs suggesting that the SGs function as a common factor and provide the scaffold to coordinate the function of the two proteins.

## Results

### Interleukin-1β induces the expression of ER stress markers, eIF2α phosphorylation and SGs assembly in human OA chondrocytes

**Figure 1:**
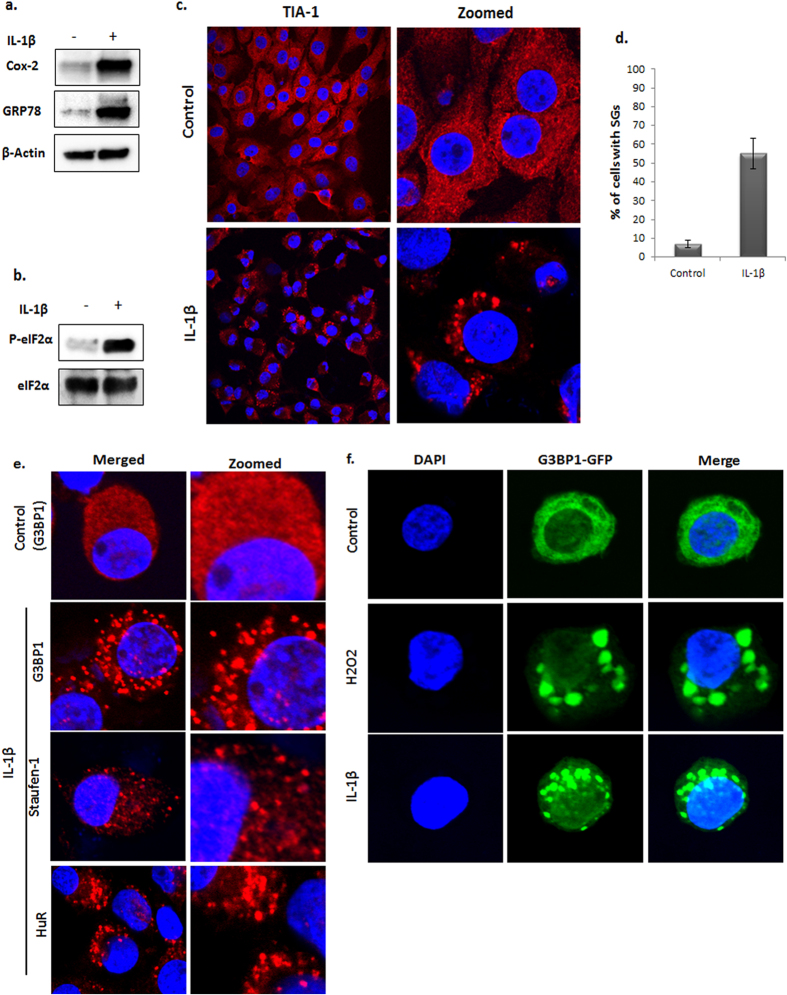
IL-1β induces the assembly of stress granules in human OA chondrocytes. (**a**) Primary human OA chondrocytes isolated from OA patients were seeded in 35-mm dishes (1 × 10^6^ OA chondrocytes/dish) for 2 to 3 days in DMEM-F12 supplemented with 10% serum. OA chondrocytes were either treated or untreated with IL-1β (10 ng/ml) and harvested after 24 hours. Cell lysate was prepared in RIPA buffer supplemented with protease and phosphatase inhibitor and expression of COX-2 and GRP78 (BiP) was analyzed by Western blotting. β-actin was used as a loading control. (**b**) OA chondrocytes were treated with IL-1β for 1 hour. The OA chondrocytes were lysed and analyzed for eIF2α phosphorylation. Total eIF2α was used as loading control. (**c**,**d**) Primary OA chondrocytes were seeded in 8-well chamber slides (0.05 × 10^6^ OA chondrocytes/well) for 2 to 3 days. The OA chondrocytes were treated with IL-1β for 1 hour and fixed with 4% paraformaldehyde (PFA) and permeabilzed in 0.3% Triton X-100. SGs were visualized by immunofluorescence staining with primary antibody specific to TIA-1 followed by Alexafluor 546 secondary antibody. The quantification of SGs is shown in Fig. 1d. (**e**) Cells were treated with IL-1β as above and probed with G3BP1, Staufen-1 and HuR followed by Alexafluor 594 secondary antibody to analyze SGs assembly. (**f**) OA chondrocytes were transfected with a G3BP1-GFP plasmid construct for 48 hours followed by treatment with Il-1β or H2O2 for 1 hour. The OA chondrocytes were fixed as above and counter-stained with DAPI to visualize the nuclei and mounted with anti-fade mounting medium. The slides were visualized with an Olympus FV1000 confocal microscope.

**Figure 2:**
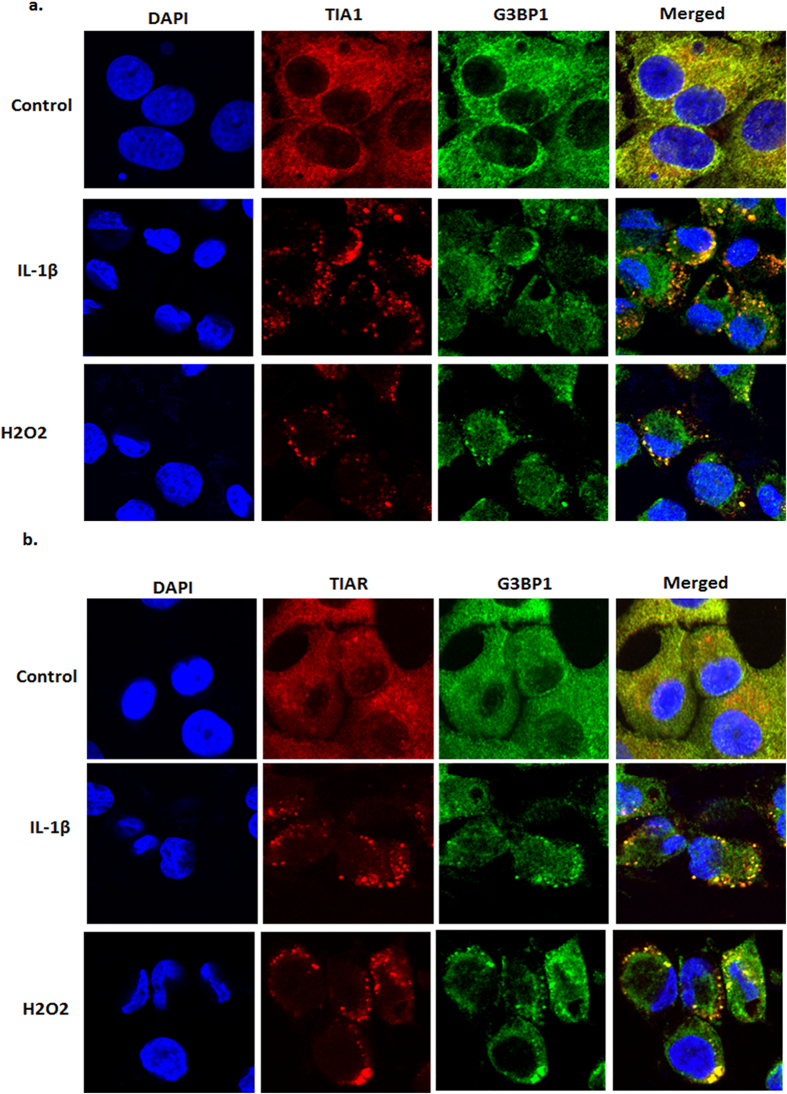
Localization of marker proteins in IL-1β induced stress granules in OA chondrocytes. Human OA chondrocytes were seeded in 8-well chamber slides (0.05 × 10^6^ OA chondrocytes/well) for 2 to 3 days and treated with either IL-1β or H_2_O_2_ for 1 hour. The OA chondrocytes were fixed, permeabilzed and immunostained with primary antibodies specific TIA-1 (mouse) and G3BP1 (rabbit) (**a**) or TIAR (goat) G3BP1 (rabbit) (**b**) followed by anti-mouse/goat AlexaFluor 546 and anti-rabbit AlexaFluor 594 secondary antibodies. The nuclei were visualized by anti-fade mounting medium containing DAPI and images were acquired using an Olympus FV1000 confocal microscope. Treatment with H_2_O_2_ was used as positive control for SGs assembly.

IL-1β is a major player in OA pathogenesis and *in vitro* studies have demonstrated that exposure to IL-1β induces ER stress as well as oxidative stress and increased expression of the matrix degrading proteases and inflammatory mediators^8^,^17^,^18^,^19^. We observed increased expression of GRP78 (Bip), a marker of ER stress, in IL-1β stimulated OA chondrocytes (Fig. 1a). Furthermore, we found that OA chondrocytes stimulated with IL-1β showed increased phosphorylation of eIF2α (Fig. 1b), which is a known trigger for the assembly of SGs. To determine whether IL-1β-induced phosphorylation of eIF2α results in the assembly of SGs in OA chondrocytes, we performed immunofluorescence staining using antibodies specific for T cell intracellular antigen-1 (TIA-1) a marker of SGs. OA chondrocytes stimulated with IL-1β showed clearly defined specks positive for TIA-1 (Fig. 1c) in more than 50% of OA chondrocytes (Fig. 1d). Assembly of SGs was further confirmed by immunofluorescence staining of GTPase activating protein (SH3 domain) binding protein 1 (G3BP1), Staufen-1 and HuR, which are RNA binding proteins known to localize to SGs^20^,^21^. As shown in Fig. 1e, IL-1β induced SGs in OA chondrocytes were enriched for G3BP1, Staufen1 and HuR proteins. Assembly of SGs in IL-1β stimulated OA chondrocytes was also confirmed by ectopic expression of GFP-tagged G3BP1 protein (Fig. 1f). OA chondrocytes treated with H_2_O_2_ were used as positive control for SGs formation in these studies. Furthermore, the assembly of SGs in OA chondrocytes was also confirmed by analyzing the colocalization of the marker proteins TIA-1 (Fig. 2a) and TIAR (Fig. 2b) with G3BP1. The protein nature of SGs in IL-1β-stimulated OA chondrocytes was confirmed by cotreatment with the protein synthesis inhibitor Cyclohexamide. As shown in Supplementary Figure 1, treatment of OA chondrocytes with Cyclohexamide prior to IL-1β addition abolished the assembly of SGs.

### SGs capture and stabilize COX-2 mRNAs in OA chondrocytes

**Figure 3:**
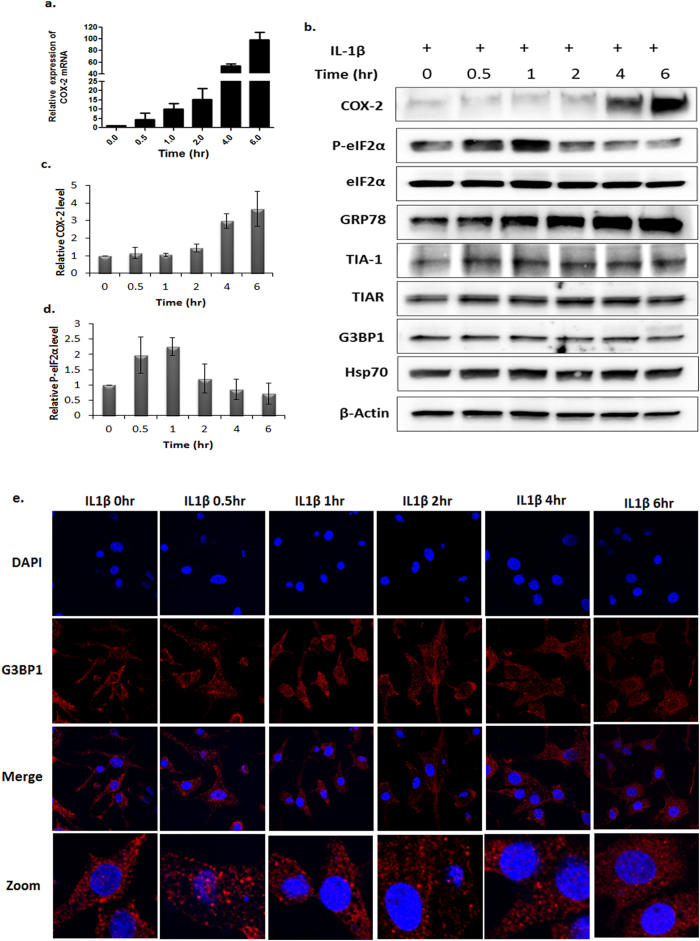
COX-2 protein expression correlates with the assembly and disassembly of SGs in OA chondrocytes. (**a**) Human OA chondrocytes were seeded in 35-mm dishes (1 × 10^6^ OA chondrocytes/dish) for 2 to 3 days and then treated with IL-1β for the indicated times. Total RNA was isolated and the COX-2 transcript level was measured by TaqMan assay for COX-2. The graph shows fold change of COX-2 mRNA relative to β-Actin. The data represent the relative levels of COX-2 mRNA from 3 patient samples (mean ± SD). (**b**) OA chondrocytes were seeded in 35-mm dish as above and treated with IL-1β for the indicated times, harvested and lysed in RIPA buffer supplemented with protease and phosphatase inhibitor. The lysate was analyzed for GRP78, COX-2, eIF2α phosphorylation and stress granule marker proteins by Western blot. (**c**) and (**d**) Densitometric analysis of COX-2 (relative to β-actin) and phospho-eIF2α (relative to eIF2α) was performed using ImageJ. The data represent the relative levels of COX-2 protein or phospho-eIF2α from 3 patient samples with the standard deviation. (**e**) OA chondrocytes were seeded in 8-well chamber slides as described in Fig. 1. The OA chondrocytes were treated with IL-1β for the indicated times, fixed with 4% PFA and permeabilized with 0.3% Triton X100-PBS. The formation of SGs was analyzed by immunofluorescence staining with anti-G3BP1 antibody.

Stress granules are ribonucleoprotein (RNP) complexes containing translationally stalled mRNAs and several RBPs. Several RBPs, e.g., TIA-1, HuR and TTP, bind to mRNAs with high ARE content and modulate their stability and translation. Since COX-2 mRNA (NM_000963) has high ARE content we determined which RBP bind and sequester COX-2 mRNAs in SGs and whether sequestration in SGs play a role in delaying COX-2 protein expression in OA chondrocytes treated with IL-1β. For these studies, OA chondrocytes were treated with IL-1β and harvested at the indicated time points and half of the OA chondrocytes were used to prepare total RNA to analyze COX-2 mRNA expression by TaqMan assay and the other half were used to prepare cell lysate to analyze the COX-2 protein levels. Stimulation of OA chondrocytes with IL-1β resulted in rapid COX-2 mRNA expression with the mRNA transcripts easily detectable as early as 30 min post-treatment and the COX-2 mRNAs level continued to increase over time (Fig. 3a). Interestingly, at the same time points there was no corresponding increase in COX-2 protein levels (Fig. 3b). An increase in the expression of COX-2 protein was observed only 4 hours post-stimulation with IL-1β (Fig. 3b,c). Analysis of GRP78 expression, a heat shock protein and marker of ER stress, was used as control because heat shock protein mRNAs are not sequestered in SGs^22^. We observed a continuous increase in the levels of GRP78 between 1 to 6 hours post-treatment with IL-1β (Fig. 3b) indicating that translation of GRP78 mRNAs was not blocked due to SGs assembly. Phosphorylation of eIF2α was also increased at 30 minutes post-IL-1β treatment and decreased after 2 hours (Fig. 3b,d) which correlated with the assembly and disassembly of SGs respectively (Fig. 3e). These results clearly demonstrate that pathological condition (stimulation with IL-1β) induces ER stress and the phosphorylation of eIF2α that triggers the assembly of SGs in human OA chondrocytes. In the present study no changes in TIA-1, TIAR or G3BP1 expression level was observed, at least up to six hours post-stimulation with IL-1β (Fig. 3b) indicating that the assembly of SGs was not due to IL-1β-induced overexpression of SGs associated proteins but rather due to the rearrangement of these proteins in the cytosol of OA chondrocytes under pathological conditions.

**Figure 4:**
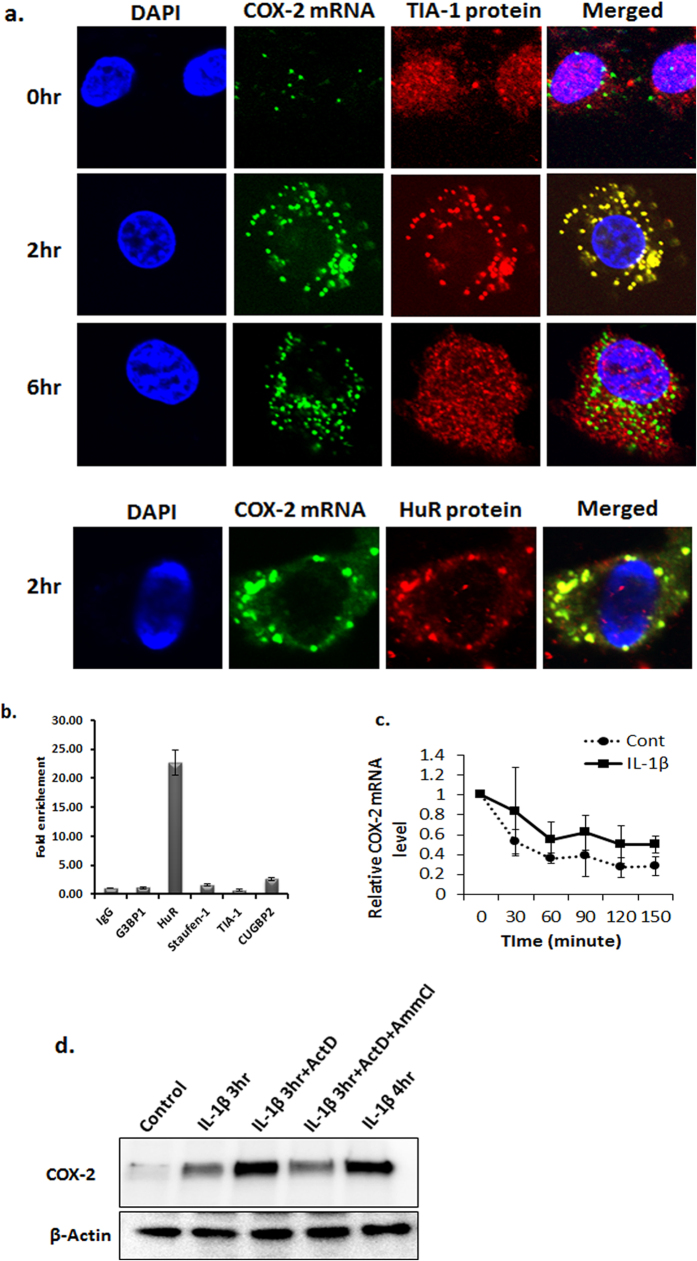
COX-2 mRNAs are sequestered in stress granules. (**a**) RNA fluorescence *in situ* hybridization (FISH) analysis of COX-2 mRNA and co-immunostaining of TIA-1 or HuR. OA chondrocytes were seeded in 8-well chamber slides as described in Fig. 1 and treated with IL-1β for indicated time. RNA FISH was performed as per the manufacturer’s protocol (RNAscope assays, Advanced Cell Diagnostics). After RNA FISH, the OA chondrocytes were probed with anti-TIA-1 or anti-HuR antibody to visualize SGs. The OA chondrocytes were mounted with antifade mounting medium containing DAPI, and images were acquired. (**b**) OA chondrocytes (50 × 10^6^ OA chondrocytes) were treated with IL-1β for 2 hours, washed extensively with PBS and lysed in polysome lysis buffer to immunoprecipitate large RNP complexes. The lysate was immunoprecipitated with rabbit-G3BP1, rabbit-HuR, rabbit-Satufen-1, mouse-TIA-1 or rabbit- CUGBP2 and mouse or rabbit IgG antibodies as control. The RNA was isolated by Trizol-chloroform extraction and analyzed for COX-2 mRNA by RT-qPCR using a TaqMan assay and each IP was normalized with Ct value from input RNA fraction. The results represent the fold enrichment of COX-2 mRNA from three independent immunoprecipitation experiments. (**c**) OA chondrocytes were seeded in 35-mm dishes as described earlier. IL-1β was added to the OA chondrocytes for 1 hour followed by the addition of actinomycin D. The OA chondrocytes were harvested at the indicated time post-actinomycin D addition. Total RNA was isolated by Trizol-chloroform extraction and the COX-2 mRNA levels were analyzed by RT-qPCR with a Taqman assay and normalized to β-Actin. (**d**) OA chondrocytes were treated with IL-1β for 3 hours followed by addition of actinomycin D or Ammonium Chloride for 1 hour. The OA chondrocytes were washed with PBS lysed in RIPA buffer and analyzed for COX-2 by immunoblotting. β-actin is used as loading control.

To determine if the delayed expression of the COX-2 protein in IL-1β-stimulated OA chondrocytes was due to the binding of COX-2 mRNAs with a specific RBP and subsequent sequestration into SGs, we determined the association of COX-2 mRNA with SGs. OA chondrocytes were treated with IL-1β for indicated time, followed by fluorescence *in situ* hybridization (FISH) of COX-2 mRNA and immunofluorescence staining for TIA-1 or HuR proteins. A granular pattern of COX-2 mRNA distribution that colocalized with the SGs marker proteins TIA-1 and HuR was clearly visible and demonstrated that COX-2 mRNA was sequestered in SGs in OA chondrocytes stimulated with IL-1β (Fig. 4a). Expression of COX-2 mRNA was barely detectable in unstimulated OA chondrocytes (Fig. 4a). Interestingly most of the COX-2 mRNA did not colocalized with TIA-1 protein at 6 hours post stimulation with IL-1β suggesting that the COX-2 mRNAs were no longer sequestered in SGs (Fig. 4a). Negative control probe did not pick any COX-2 mRNA staining demonstrating the specificity of the COX-2 mRNA FISH performed (RNAScope, Supplementary Figure 2) We next determined the protein partner that bind the COX-2 mRNAs by RNA immunoprecipitation-Chip assay developed by Keene *et al*.^23^ for immunoprecipitation of high molecular weight RNP complexes using antibodies specific for G3BP1, HuR, Staufen-1, TIA-1 and CUGBP2 proteins. We found highest enrichment of COX-2 mRNAs in HuR immunoprecipitates, which indicated that COX-2 mRNA was principally captured and localized to the SGs by the HuR protein (Fig. 4b and Supplementary Figure 3). Binding of COX-2 mRNAs by HuR promotes COX-2 mRNA stability and translation in other cell types^24^ but has not been shown in OA chondrocytes. We therefore analyzed the effect of COX-2 mRNA sequestration in SGs on its stability by performing Actinomycin D chase experiment. We treated OA chondrocytes with IL-1β for 1 hour to allow the assembly of SGs, followed by addition of Actinomycin D, then OA chondrocytes were harvested at indicated times for total RNA preparation and COX-2 mRNA level determination by TaqMan assay. Our results demonstrated an increase in the half-life of COX-2 mRNA in OA chondrocytes under conditions and durations of treatment that promoted SGs assembly (Fig. 4c). We also analyzed whether the COX-2 mRNA associated with SGs is directed for degradation or translation after the SGs were dissolved. To test this, we treated OA chondrocytes with IL-1β for 3 hours followed by the addition of Actinomycin D to prevent further transcription. The OA chondrocytes were harvested after 4 hours of IL-1β treatment or IL-1β + Actinomycin D treatment and analyzed for COX-2 protein levels by immunoblotting. COX-2 protein level in OA chondrocytes treated with Actinomycin D after 3 hours of IL-1β treatment was similar to COX-2 protein level in OA chondrocytes treated for 4 hours with IL-1β indicating that COX-2 mRNA associated with SGs was directed for translation (Fig. 4d). As control, we added Ammonium Chloride with Actinomycin D to prevent the dissolution of SGs and analyzed COX-2 protein levels. COX-2 protein levels in OA chondrocytes treated with IL-1β + Actinomycin D + Ammonium Chloride were similar to the levels in OA chondrocytes treated with IL-1β alone for 3 hours. These findings support the hypothesis that IL-1β induced COX-2 mRNA is sequestered into SGs and remains stable during this period. Importantly, these data demonstrate that the delay in the appearance of COX-2 protein in IL-1β stimulated OA chondrocytes was associated with the sequestration of COX-2 mRNA in SGs which blocked its translation.

### Inhibition of SGs clearance dampens COX-2 expression

**Figure 5:**
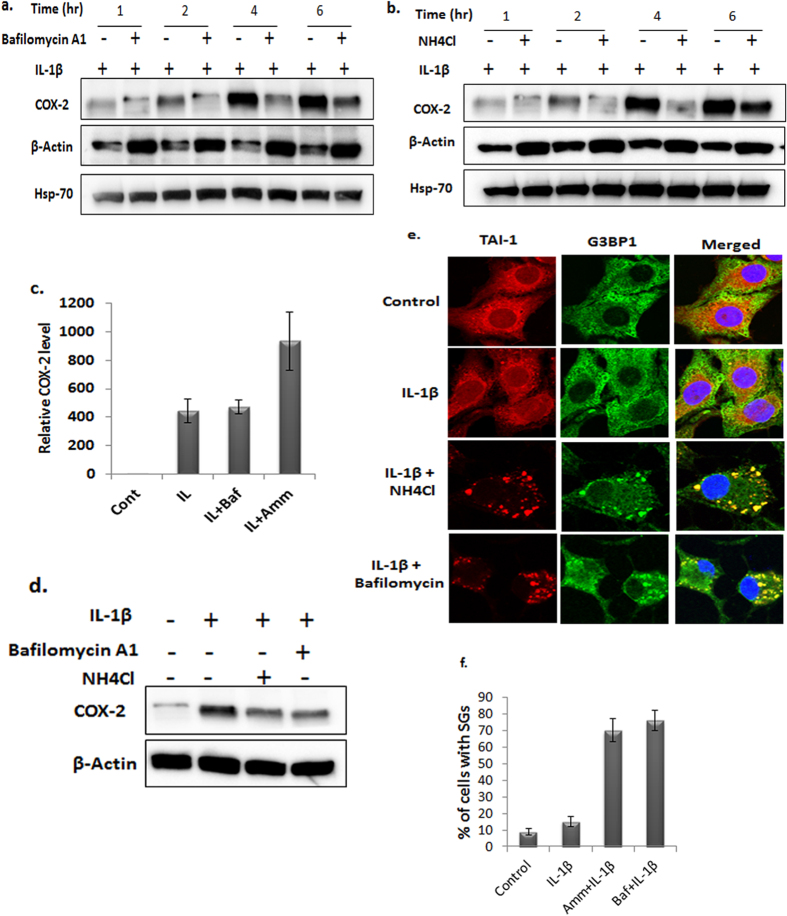
Inhibition of autophagy block stress granule’s clearance and dampens COX-2 expression. OA chondrocytes were seeded in 35-mm dishes as described earlier. The OA chondrocytes were pretreated with either Bafilomycin A1 (**a**) or Ammonium Chloride (**b**) for 2 hours followed by the addition of IL-1β for the indicated times. The OA chondrocytes were washed with PBS and lysates were prepared in RIPA buffer supplemented with protease inhibitor and analyzed by western blotting for COX-2 protein. (**c**) Total RNA was isolated from OA chondrocytes pretreated with Bafilomycin A1 or Ammonium Chloride followed by IL-1β for 6 hours. COX-2 transcripts were quantitated by RT-qPCR with TaqMan assay and normalized to β-Actin. (**d**) The OA chondrocytes were first treated with IL-1β for 1 hour and then Bafilomycin A1 or Ammonium Chloride was added. The OA chondrocytes were harvested after 6 hours, and the lysate was analyzed for COX-2 expression by western blotting. (**e**,**f**) The OA chondrocytes were seeded in 8-well chamber slides as described in Fig. 1 and treated with either bafilomycin A1 or Ammonium Chloride for 2 hours followed by IL-1β for 6 hours. The OA chondrocytes were fixed in 4% PFA and immunostained with mouse-TIA-1 and rabbit-G3BP1 primary antibodies and anti-mouse AlexaFluor 546 and anti-rabbit AlexaFluor 594 secondary antibodies. The OA chondrocytes were mounted in antifade mounting medium containing DAPI, and images were acquired using an Olympus FV1000 confocal microscope. The graph shows the quantitation of SGs.

After overcoming the stress by the cell, SGs are cleared by lysosomal degradation mediated by the autophagy pathway^25^. Therefore, we hypothesized that if SGs clearance is blocked, this will result in delayed translation of the sequestered COX-2 mRNAs. To test this we blocked SGs clearance by Bafilomycin A1, an inhibitor of autophagosome fusion with lysosome. Treatment of OA chondrocytes with Bafilomycin A1 blocked the SGs clearance and significantly delayed the expression of COX-2 protein in OA chondrocytes under pathological condition (Fig. 5a and Supplementary Figure 4A). Treatment of OA chondrocytes with Ammonium Chloride, an inhibitor of lysosomal function, essentially provided results similar to those obtained with Bafilomycin A1 treatment (Fig. 5b and Supplementary Figure 4B). Notably, we did not observe any decrease in COX-2 transcripts level upon treatment with either Bafilomycin A1 or Ammonium Chloride (Fig. 5c), which indicated that the nearly absent COX-2 protein in IL-1β stimulated OA chondrocytes was due to the translational repression of its mRNA due to sequestration in SGs. Furthermore, to eliminate the possibility that the reduced levels of COX-2 protein were due to Bafilomycin A1 or Ammonium Chloride interference with IL-1β signaling, we pretreated the OA chondrocytes with IL-1β followed by the addition of Bafilomycin A1 or Ammonium Chloride and obtained essentially the same results (Fig. 5d). We confirmed the presence of SGs in OA chondrocytes with autophagy blockage after 6 hours of IL-1β treatment by immunofluorescence staining of the SGs marker proteins G3BP1 and TIA-1 (Fig. 5e,f). Taken with above findings, these results suggest that the delayed expression of COX-2 protein was not due to the inhibition of IL-1β signaling but due to the translational repression of COX-2 mRNAs. These results indicate that sequestration of COX-2 mRNA in SGs delayed its translation in human OA chondrocytes at least during the period of the inflammatory response analyzed when the OA chondrocytes were under enhanced stress due to the activation of several signaling pathways and rapid production of inflammatory mediators.

### Abolition of SGs assembly causes rapid expression of COX-2 protein in OA chondrocytes

**Figure 6:**
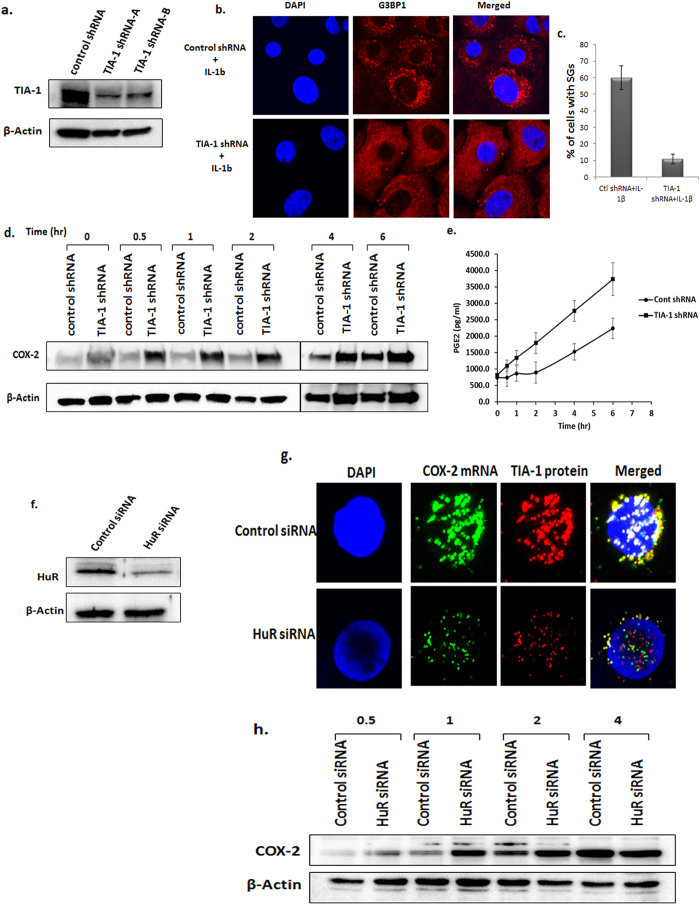
Depletion of TIA-1 or HuR expression accelerates COX-2 mRNA translation in OA chondrocytes. (**a**) OA chondrocytes (1 × 10^6^) were transfected with control or TIA-1 shRNA plasmid constructs. After 48 hours, the OA chondrocytes were harvested and analyzed for TIA-1 knockdown by western blotting using anti-TIA-1 antibody. (**b,c**) OA chondrocytes were transfected with control or TIA-1 shRNA and seeded in 8-well chamber slides for 48 hours followed by IL-1β treatment for 1 hour. The OA chondrocytes were washed with PBS, fixed in 4% PFA and immunostained with G3BP1 as described in Fig. 1. (**d**) OA chondrocytes were transfected with control or TIA-1 shRNA for 48 hours and treated with IL-1β for different times. Cell lysate was prepared in RIPA buffer and analyzed for COX-2 expression by western blotting. β-actin is used as loading control. (**e**) The supernatant from the above experiment was collected and analyzed for PGE2 using an ELISA kit from R&D systems. (**f**) OA chondrocytes (1 × 10^6^) were transfected with control or HuR siRNA (50 nM) and incubated for 48 hours. The OA chondrocytes were harvested and analyzed for HuR knockdown by western blotting. (**g**) Chondrocytes were transfected with either control or HuR siRNA for 48 hours followed by treatmetn with IL-1β for 2 hours. RNA FISH for COX-2 mRNA and protein immunofluorescence staining for TIA-1 was done as in Fig. 4. (**h**) OA chondrocytes were transfected with control or HuR siRNA for 48 hours and treated with IL-1β for different times. Cell lysate was prepared in RIPA buffer and analyzed for COX-2 expression by western blotting. β-actin is used as loading control.

TIA-1, an RNA binding protein is an essential component and required for the assembly of SGs^26^. Results shown above demonstrated that by blocking SGs clearance, repression of COX-2 mRNA can be extended significantly. This suggested that if the assembly of SGs can be blocked, this will result in early and rapid translation of COX-2 mRNA. To test this we first standardized the shRNA-mediated depletion of the TIA-1 expression in OA chondrocytes (Fig. 6a) and then used the same shRNAs to transfect the OA chondrocytes which were then stimulated with IL-1β for different time periods. Depletion of TIA-1 resulted in loss of SGs assembly in response to IL-1β treatment in OA chondrocytes (Fig. 6b,c). In time kinetics experiments using TIA-1-depleted OA chondrocytes we demonstrate that the COX-2 protein expression was very early and increased significantly compared with OA chondrocytes transfected with control shRNA. The increase in COX-2 protein expression started as early as 30 minutes post IL-1β treatment of OA chondrocytes while in control, COX-2 protein expression was detected after 4 hours post stimulation with IL-1β (Fig. 6d and Supplementary Figure 5). We also quantified the production of PGE_2_ in the supernatants of control and TIA-1 depleted OA chondrocytes and found that PGE_2_ production essentially followed the expression pattern of COX-2 protein, wherein its production was linear in TIA-1 depleted cells but was delayed in control shRNA transfected OA chondrocytes (Fig. 6e). Furthermore, to confirm that HuR protein binds and sequester COX-2 mRNAs in SGs in human OA chondrocytes, siRNA mediated knockdown of HuR expression was employed. siRNA-mediated knockdown of HuR (Fig. 6f) affected the sequestration of COX-2 mRNA into SGs without compromising SGs assembly in cytokine-stimulated OA chondrocytes (Fig. 6g). Also HuR depletion resulted in early and increased expression of COX-2 protein (Fig. 6h and Supplementary Figure 6) suggesting that in the absence of sequestration of COX-2 mRNAs in SGs, translation of COX-2 mRNAs proceeds unhindered. These results further support our hypothesis that the observed delay in the COX-2 protein expression was due to the sequestration and stalling of COX-2 mRNAs translation in SGs in OA chondrocytes under pathological conditions.

## Discussion

Treatment of OA chondrocytes with the pro-inflammatory cytokine IL-1β, a major player in OA pathogenesis, initiates a cascade of signaling events that leads to the activation of nuclear factor kappa B (NFκB) and other transcription factors^27^,^28^ culminating in the induction and expression of the catabolic factors and downregulation of anabolic factors^29^. IL-1β induces the expression of COX-2 and production of PGE_2_ in chondrocytes but there is a significant delay between the mRNA expression and protein expression^5^,^8^. In the present study, we investigated the mechanism of delayed COX-2 protein expression in IL-1β stimulated OA chondrocytes. We report here that IL-1β treatment induce ER stress in human OA chondrocytes that leads to the increased expression of the GRP78 chaperone protein and phosphorylation of eIF2α. In time dependent stimulation of OA chondrocytes with IL-1β, we observed a dynamic pattern of eIF2α phosphorylation (Fig. 3b) and the phosphorylation dynamics of eIF2α coincided with the dynamics of SGs formation and dissolution in OA chondrocytes (Fig. 3e). This is also the first report to demonstrate that IL-1β induces SGs assembly in human OA chondrocytes. SGs are RNP complexes formed under cellular stress conditions and have been suggested to play important roles in the posttranscriptional regulation of selected population of mRNAs^20^,^30^. A study by Moeller *et al*.^31^ has shown that the expression of downstream targets of hypoxia inducible factor-1α (HIF1α) is delayed due to the formation of SGs in cells. Similar observations have been found in an animal model of ischemia, in which the assembly of SGs blocked mRNA translation in neurons^32^. Of interest is our finding that COX-2 mRNA levels increased significantly within 1 hour of IL-1β treatment but the COX-2 protein levels increased only after the disassembly of SGs. These results are indicative of the translational repression of COX-2 mRNA possibly due to sequestration by SGs in OA chondrocytes under pathological conditions. We found that the expression of GRP78, an ER molecular chaperone, increases in a time-dependent manner in response to IL-1β treatment, because heat shock protein mRNAs do not package in SGs. Our results demonstrate that SGs are important players in the posttranscriptional regulation of COX-2 (or other associated mRNAs) expression in OA chondrocytes under pathological conditions.

Autophagy, an evolutionarily conserved phenomenon, plays a major role in the clearance of SGs, and the inhibition of autophagy reduces SGs clearance^25^. To support our hypothesis that the delay in COX-2 protein expression was probably due to the sequestration of IL-1β induced COX-2 mRNA in SGs and suppression of its translation, therefore a delay in SGs dissolution should coincide with the delayed translation of COX-2 mRNA. OA chondrocytes with blocked autophagy failed to clear SGs formed after IL-1β stimulation and inhibited COX-2 protein expression for significantly longer duration than OA chondrocytes without autophagy blockage (Fig. 5a,b and Supplementary Figure 4). Interestingly treatment of chondrocytes with Bafilomycin A1 prolonged the phosphorylation of eIF2α (data not shown) suggesting that the increased persistence of SGs was due to prolonged eIF2α activation. Lysosomal function inhibition by Ammonium Chloride also resulted in the increased duration of SGs presence in OA chondrocytes and delayed COX-2 protein expression. However, Ammonium Chloride or Bafilomycin A1 did not affect COX-2 mRNA expression levels, confirming that the low COX-2 protein level in OA chondrocytes stimulated with IL-1β was due to the translational repression of COX-2 mRNA.

Association of an mRNA with a particular RBP depends on the type as well as condition of the cell and decides the fate of the transcript. The fate of the mRNAs sequestered in SGs is not very well understood. Once the stress is resolved and SGs are cleared, the mRNAs captured in SGs can function as templates for polysome assembly and translation, or the mRNAs can also be targeted to processing bodies for degradation^20^,^22^. A study by Dixon *et al*. has shown that TIA-1 knockout fibroblasts express a high amount of COX-2^33^. Also defective binding of TIA-1 to COX-2 mRNA has been reported to increase COX-2 expression in colon cancer cells^33^. TIA-1 knockout mice have elevated TNFα and COX-2 levels and develop mild arthritis suggesting its importance in the development of inflammatory arthritis, however, the mechanism behind the gene regulation is not known^14^. We found that TIA-1 knockdown abrogated SGs assembly in OA chondrocytes stimulated with IL-1β. Additionally, in OA chondrocytes with TIA-1 knockdown early and increased COX-2 protein and PGE2 expression levels were observed in response to IL-1β treatment. We also found that COX-2 mRNA was principally associated with the RNA binding protein HuR in SGs and not with TIA-1. However, TIA-1 knockdown abrogated SGs assembly and resulted in increased COX-2 protein expression levels in OA chondrocytes under pathological conditions. Depletion of HuR protein in OA chondrocytes did not abrogate the assembly of SGs in human OA chondrocytes but it abolished the sequestration of COX-2 mRNAs in SGs (Fig. 6g). In addition, siRNA mediated depletion of HuR expression also resulted in early and increased expression of COX-2 protein (Fig. 6h and Supplementary Figure 6) further highlighting the role of HuR protein in recruitment and translational suppression of COX-2 mRNA via sequestration in SGs. These data further demonstrate that although TIA-1 protein was not directly associated with COX-2 mRNAs but that it may be an important player in the repression of COX-2 mRNAs translation through the assembly of SGs. Furthermore, these results provided unique information about the coordinated function of the RBPs in gene regulation and that assembly of SGs provide scaffold for protein interaction and function. Our findings imply that deletion or functional impairment of any of the genes that are essential for SGs assembly may enhance the induction and progression of OA.

Our results also suggest that the assembly of SGs in OA chondrocytes is a critical event that suppresses COX-2 protein levels and functions as a cellular defense mechanism against IL-1β-induced stress. Thus, in our opinion, SGs represent a novel target for developing therapies to suppress COX-2 protein expression. It will also be important to study the role of SGs on the expression of other genes involved in OA pathogenesis. The mechanism by which IL-1β regulates eIF2α phosphorylation and the dynamics of SGs assembly requires further exploration. Furthermore, the signaling events or the players responsible for SGs assembly and or dissolution might also serve as therapeutic targets in OA. These observations highlight the interesting issue of posttranscriptional regulation of catabolic genes expression in OA that requires further study with a view to develop better and more effective therapies for the management of OA.

## Methods

### Human cartilage samples and Preparation of primary human chondrocytes

Prior to the initiation of the studies the study protocol was reviewed and approved by the Institutional Review Board (IRB) of Northeast Ohio Medical University, Rootstown, Ohio as a “non-human subject study under 45 CFR” and that no informed consent was needed. All the methods used in this study were carried out in accordance with the approved guidelines and all experimental protocols were approved by the IRB of Northeast Ohio Medical University, Rootstown, Ohio.

Discarded and de-identified knee or hip joint cartilage samples were collected through the NIH supported National Disease Research Interchange (NDRI) per the IRB approved protocol. The unaffected areas of the cartilage were used to prepare OA chondrocytes as described previously^34^.

### Culture medium and other reagents

For OA chondrocyte culture, DMEM-F-12 (#12-719Q) was procured from Lonza (Walkersville, MD, USA), fetal calf serum (#10437028), Actinomycin D (A7592) and other cell culture reagents were purchased form Life Technologies (Carlsbad, CA, USA). Pronase (#11459643001) and Collagenase (#11088793001) were from Roche Diagnostics (Indianapolis, IN, USA). Recombinant human IL-1β and the PGE_2_ ELISA kit (#201-LB-025 and # KGE004B respectively) were purchased from R&D Systems (St Paul, MN, USA). Antibodies against β-Actin (sc-47778), TIA-1 (sc-365349), TIAR (sc-1749), G3BP1 (sc-98561), Hsp-70 (sc-24), HuR (sc-20694) and Staufen-1(sc-134042) were from Santa Cruz Biotechnology (Santa Cruz, CA, USA). Antibodies against GRP78 (C50B12), eIF2α (#9722) and phospho-eIF2α (#9721) were from Cell Signaling Technology (Beverly, MA, USA) and anti-COX-2 antibody (AF4198) was from R&D Systems. Horseradish peroxidase conjugated appropriate secondary antibodies were from Cell Signaling Technology (#7074) or Santa Cruz Biotechnology (sc-2020) or Thermo Scientific (31432).

### Immunofluorescence staining

Primary OA chondrocytes (0.1 × 10^6^) were seeded in an 8-well chamber slide (Nunc Lab-Tek) for 2 to 3 days after isolation and treated with 10 ng/ml of IL-1β for the indicated times. The OA chondrocytes were fixed in 4% paraformaldehyde and permeabilized with 0.3% Triton X-100 containing phosphate-buffered saline (PBS) and were probed with primary and secondary antibodies as described in the text and figure legends. The slides were mounted with anti-fade medium containing DAPI (Vectashield, Vector Laboratories, Burlingame, CA, USA), and images were acquired with an Olympus FV1000 confocal microscope using a 60X oil immersion lens.

### RNA fluorescence *in situ* hybridization (FISH)

COX-2 mRNA fluorescence *in situ* hybridization was performed using FISH probes (#406801) and the RNAscope kit as per manufacturer’s instructions (Advanced Cell Diagnostics, Hayward, CA, USA). Briefly, primary human OA chondrocytes were seeded in an 8-well chamber slide as described above. The OA chondrocytes were treated with 10 ng/ml IL-1β for 2 hours and fixed in 10% neutral buffered formalin, washed with PBS, dehydrated and rehydrated with a gradient of ethanol. Finally, the OA chondrocytes were probed with the COX-2 mRNA FISH probes, washed, stained with DAPI and mounted using anti-fade medium. Images were acquired as described above.

### RNA immunoprecipitation (RIP) and qPCR

For RNA immunoprecipitation^23^ OA chondrocytes were seeded in 100 mm dishes (5 × 10^6^ OA chondrocytes/dish, total 10 dishes) and treated with 10 ng/ml IL-1β for 2 hours. In brief, OA chondrocytes were washed with PBS after IL-1β treatment and lysed using polysome lysis buffer (100 mM KCl, 5 mM MgCl_2_, 10 mM HEPES, 0.5% NP40, 1 mM DTT, 100 units/ml RNasin, 400 μM VRC and protease inhibitor cocktail) and the lysate was cleared by centrifugation. The cleared lysate was incubated with the target or control antibody in the presence of 20 mM EDTA for 12 to 18 hours followed by the addition of protein A-BSA slurry and incubated for 4 hours at 4 °C. Agarose beads were washed with NT-2 buffer (50 mM Tris-HCl, pH 7.4, 150 mM NaCl, 1 mM MgCl_2_ and 0.05% NP-40), and the RNA was isolated by Trizol-chloroform extraction. One microgram of RNA was used to prepared cDNA using the High-Capacity cDNA Reverse Transcription Kit (catalog no 4368813, Life Technologies). COX-2 mRNA levels were quantified using TaqMan gene expression assay (#HS00153133-m1, Applied Biosystems) and normalized to β-Actin expression levels.

## Additional Information

**How to cite this article**: Ansari, M. Y. and Haqqi, T. M. Interleukin-1β induced Stress Granules Sequester COX-2 mRNA and Regulates its Stability and Translation in Human OA Chondrocytes. *Sci. Rep*. **6**, 27611; doi: 10.1038/srep27611 (2016).

## Supplementary Material

Supplementary Information
