# Supplementary material for: Interleukin-1β induced Stress Granules Sequester COX-2 mRNA and Regulates its Stability and Translation in Human OA Chondrocytes
Source: Sci Rep. 2016 Jun 8;6:27611. doi: 10.1038/srep27611 (PMC4897887; doi:10.1038/srep27611)
Supplement: Supplementary Information [file srep27611-s1.pdf]

Interleukin-1β induced Stress Granules Sequester COX-2 mRNA and Regulates its Stability and Translation in Human OA Chondrocytes

Mohammad Y Ansari and Tariq M Haqqi*

Department of Anatomy and Neurobiology, North East Ohio Medical University, Rootstown, Ohio, USA

*Corresponding author: Dr Tariq M Haqqi, Department of Anatomy and Neurobiology, North East Ohio Medical University, 4209 State Route 44, Rootstown, OH 44272, USA

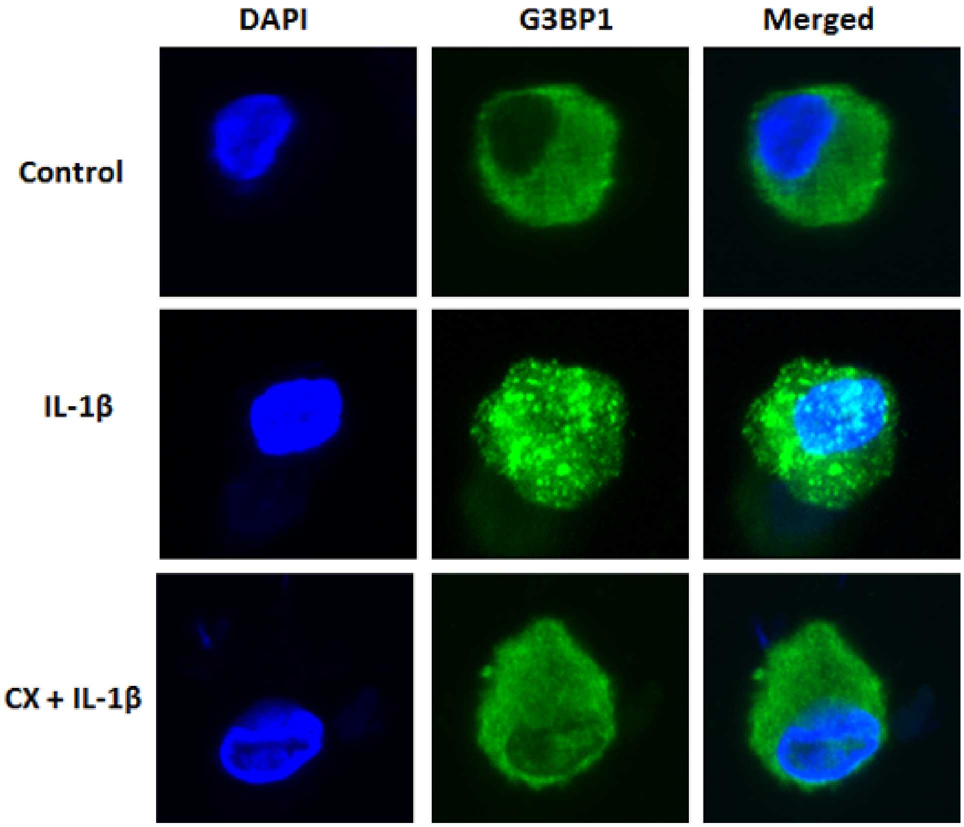

Supplementary Figure 1. Inhibition of protein synthesis block the assembly of SGs. OA chondrocytes were seeded in 8 well chamber slide as in Figure 1 and treated with Cycloheximide (CHX) prior to IL-1β addition. OA chondrocytes were fixed, permeabilized and probed with anti-G3BP1 antibody to visualize SGs assembly.

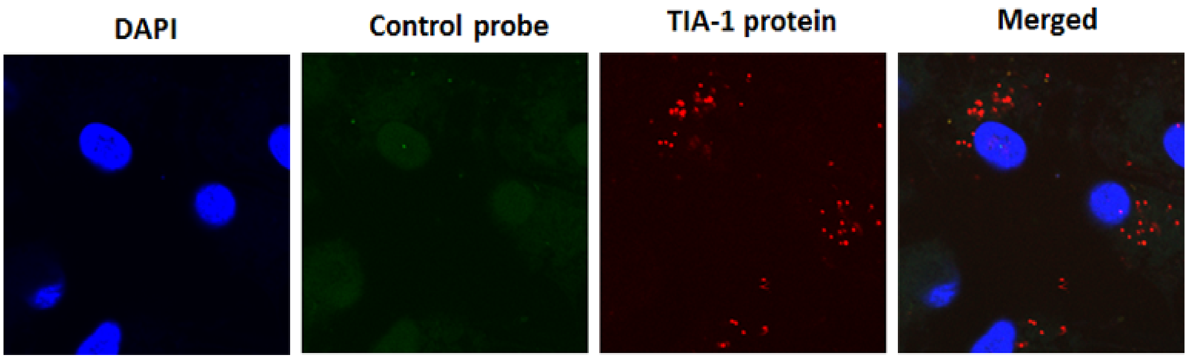

Supplementary Figure 2. Specificity of COX-2 mRNA FISH using RNAScope. OA chondrocytes were seeded in 8 well chamber slide and treated with IL-1β addition as in Figure 4 and probed with control for RNA FISH and TIA protein.

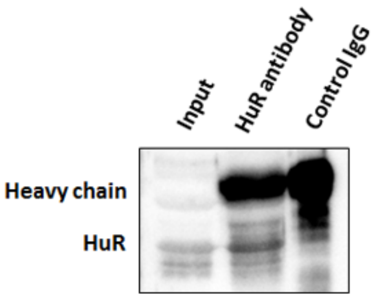

Supplementary Figure 3. Western blot analysis of HuR protein in immunoprecipitation. HuR was immunoprecipitated as described for Figure 4b in the text and analyzed by Western blotting of the immunoprecipitate. Normal Rabbit IgG was used as control.

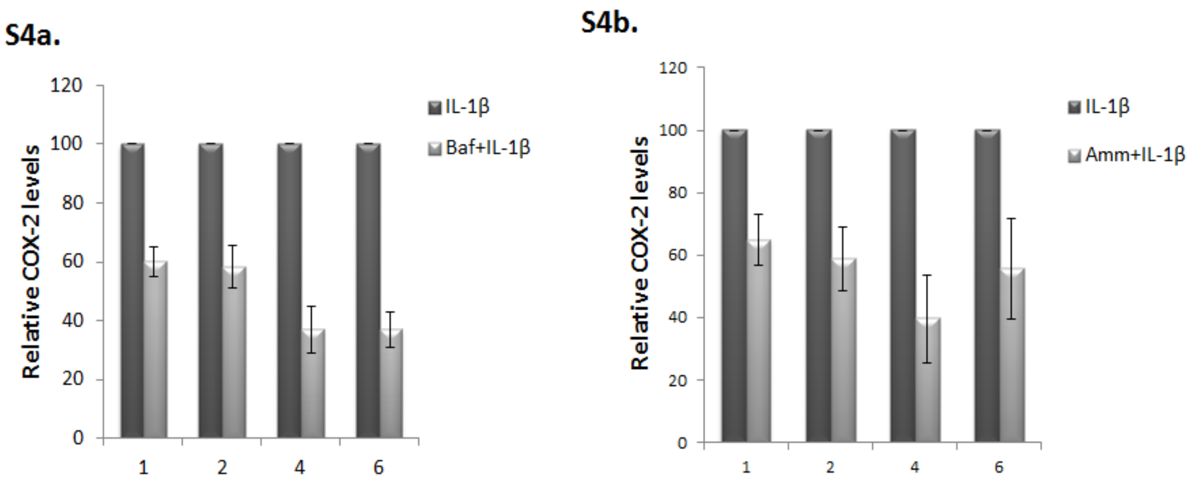

Supplementary Figure 4. Quantitation of COX-2 protein from Figure 5a (S3A) and 5b (S3B) was done using ImageJ. The values are normalized with actin and are presented as percentage of control.

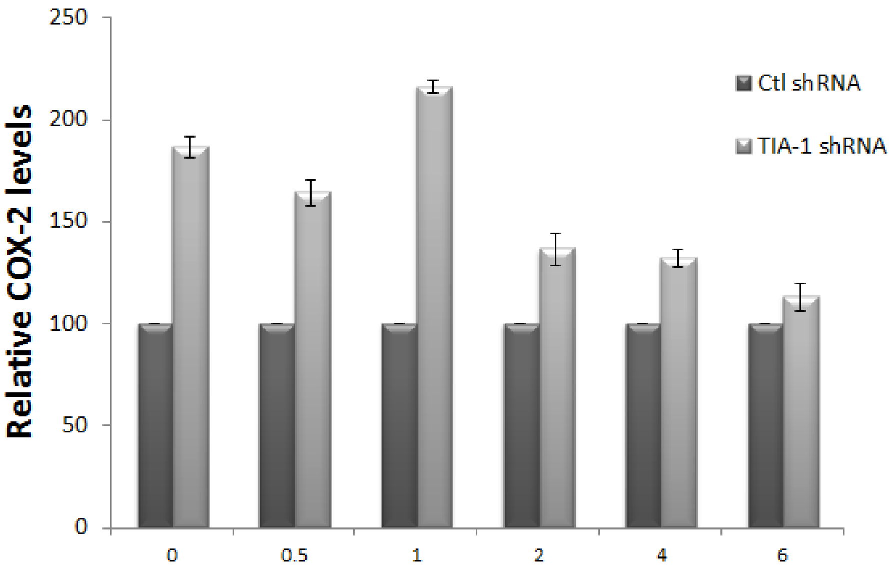

Supplementary Figure 5. Quantitation of COX-2 protein from Figure 6d was done using ImageJ. The values are normalized with actin and are presented as percentage of control.

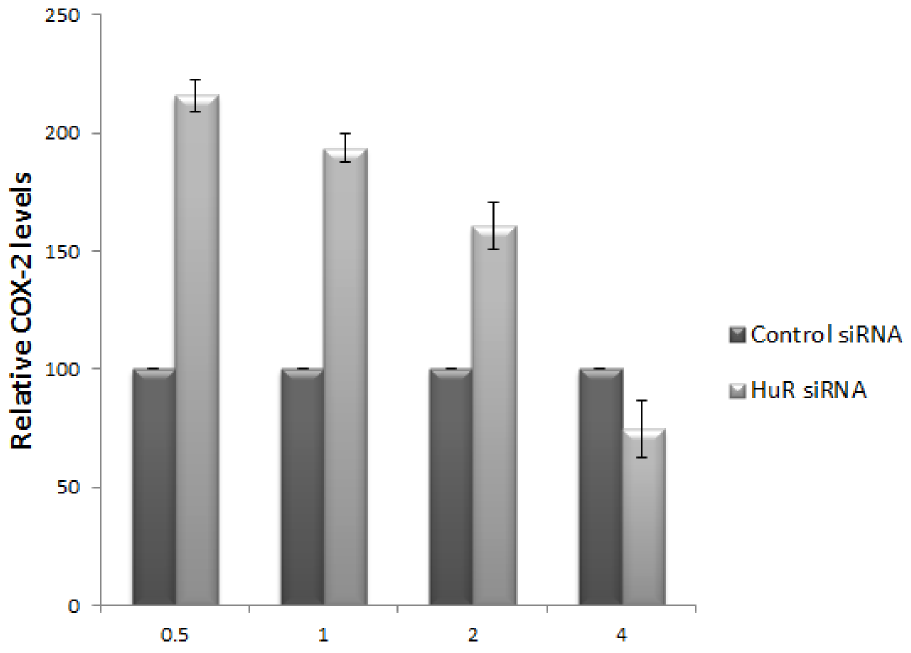

Supplementary Figure 6. Quantitation of COX-2 protein from figure 6h was done using ImageJ. The values are normalized with actin and are presented as percentage of control.
